# Evacuation of Refugees from Afghanistan: Health Control at the Spanish Border in the Context of the COVID-19 Pandemic

**DOI:** 10.3390/ijerph20075292

**Published:** 2023-03-28

**Authors:** Miguel Dávila, Patricia López, Maria Ramiro-Gonzalez, Ana M. Saénz de Urturi, Rocío del Pilar Palmera, Concepción Sánchez, Irene G. M. Wijers, Iratxe Moreno, Fernando Riesco, Lourdes Oliva, Sergio Béjar, Inmaculada Vera, Gloria González, Fernando Carreras, Pilar Aparicio

**Affiliations:** 1 Deputy Directorate of Foreign Health, Ministry of Health of Spain, 28014 Madrid, Spain; 2 Directorate General for Public Health, Ministry of Health of Spain, 28014 Madrid, Spain; 3 Department Office of Citizen Attention, Madrid City Council, 28044 Madrid, Spain; 4 National Institute of Social Security (NISS), Ministry of Inclusion, Social Security and Migration, 11011 Cádiz, Spain

**Keywords:** evacuation, Afghanistan, COVID-19, health control, point of entry, public health emergency

## Abstract

Following the announcement of the retreat of troops from Afghanistan, the Spanish Government organised the so-called “Antigone Operation” for the evacuation of Afghan collaborators. The most relevant ministries were involved in the response. The Ministry of Health, through the Foreign Health Department, performed the health control on arrival. The whole operation was conducted at an air base. It included the health control of refugees composed of temperature measurement, a basic visual control and a coronavirus disease (COVID-19) rapid antigen test for those over 12 years of age; the assessment of their basic needs (food and hygiene); identification and security procedures; and the initial administrative processing. The refugees were accommodated in a temporary facility at the base, where they waited to be transferred to their final destinations. Between 19 and 27 August 2021, 2168 refugees arrived on 17 flights; 680 of them were children under 12 years of age. One thousand four hundred and ninety-nine rapid antigen tests were performed, with one positive result. “Antigone Operation” is unprecedented in Spain and is one of the most complex operations carried out in recent years. The COVID-19 pandemic required the establishment of a health control system on arrival, performed by Foreign Health, which contributed significantly to the overall success of the operation.

## 1. Introduction

The announcement of the withdrawal of the United States (US) troops from Afghanistan on 30 August 2021 and the foreseeable return of the Taliban to power, triggered thousands of people to try to leave the country. From that moment on, many countries, including Spain, started to work on the repatriation of their own personnel and their Afghan collaborators and their families. The so-called “Antigone Operation” was based on the commitment of the Spanish Government and took place in August 2021, with the participation of the competent authorities from the different ministries, which were involved in the response in one way or another.

The “Antigone Operation” is an excellent example of a coordinated response given by the main Spanish ministries to deal with a difficult situation that arose unexpectedly. It was one of the most complex operations carried out in Spain in recent years as, in addition to the difficulties inherent in this type of intervention, the coronavirus disease (COVID-19) pandemic required the implementation of an “ad hoc” health control system upon arrival. The health response was organised and coordinated by Foreign Health, which is the competent public health authority at Spanish borders.

Spain is a decentralised country in terms of health care, but Foreign Health continues to be an exclusive competence of the State in accordance with the Spanish Constitution [1]. It is the national organisation responsible for the surveillance and control of health risks arising from international travellers, as well as for coordinating the national administrations and agencies involved in the response to events that may pose a public health risk at Spanish borders [2,3].

Foreign Health ensures compliance with the World Health Organisation’s International Health Regulations 2005 at Spanish points of entry, which requires states to have the capacity to respond to events that may constitute a public health emergency of international concern [4].

Foreign Health activities are planned and coordinated from the central level at the Deputy Directorate of Foreign Health (DDFH), Directorate General for Public Health and Ministry of Health and are implemented by the Foreign Health Control Units (FHCUs) located at international points of entry. The FHCUs are composed of medical doctors, nurses, veterinarians and pharmacists and are attached to the Ministry of Territorial Policy through the Delegations of the Government in the Autonomous Communities. This way, the FHCUs are structurally integrated into the Ministry of Territorial Policy, but they follow the instructions provided by the Ministry of Health.

On the other hand, Foreign Health is the first responder to public health events at points of entry, but once travellers have crossed the border, the competence for health care and follow-up belongs to the regional health authorities of the Autonomous Communities. Therefore, fluent coordination between Foreign Health and the Autonomous Communities is needed at all times.

This article describes the health actions carried out by Foreign Health as part of the global response given by the Spanish Government for the transfer, reception and initial welcome of refugees evacuated from Afghanistan.

## 2. Materials and Methods

### 2.1. General Description of the Operation

**Figure 1:**
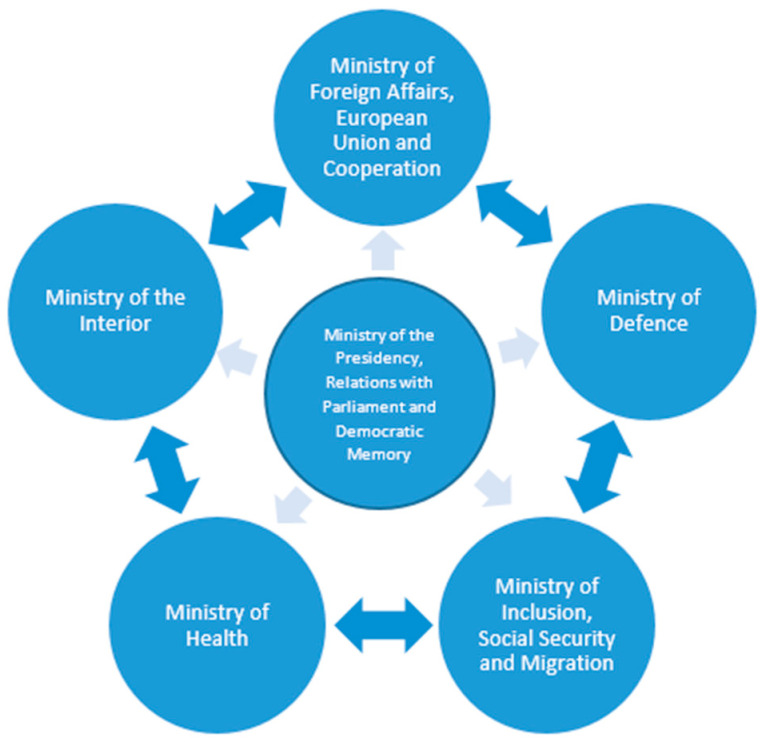
Inter-Ministerial Coordination Commission.

One of the first measures adopted by the Government was the creation of an Inter-Ministerial Coordination Commission (Figure 1) led and coordinated by the Minister of the Presidency, Relations with the Parliament and Democratic Memory. It was made up of representatives from all the ministries involved in the response: Ministry of Foreign Affairs, European Union and Cooperation; Ministry of Defense; Ministry of Inclusion, Social Security and Migration; Ministry of the Interior; and Ministry of Health. The Commission was permanently active throughout the whole operation and met daily to analyse the information received, plan every day’s flights and take the appropriate decisions in response to the problems identified.

The Ministry of Foreign Affairs, European Union and Cooperation, through the Spanish Embassy in Kabul, estimated the initial number of Afghan refugees to be evacuated at 1200. In addition and due to the commitment acquired by the Government with the European Union (EU) and the North Atlantic Treaty Organization (NATO), Spain assumed to temporarily accommodate a number of people who had collaborated with those organisations and whose final destinations were other countries, acting as a single point of entry and performing the health and the administrative controls. For this reason, the total estimated number of refugees to be controlled reached 2200.

The Ministry of Defense established a permanent airlift between Dubai and Kabul, with two military aircraft regularly flying to Kabul to pick up the refugees and transfer them to Dubai. In Dubai, the refugees were duly identified and underwent an initial health assessment by the military doctors before travelling on commercial flights specially chartered for the occasion to an air force base located in Torrejón de Ardoz, Madrid (Spain). Furthermore, some other flights carrying EU and NATO Afghan collaborators flew from Afghanistan to the base with stopovers in different European airports such as Paris, Athens and Rome. We chose the air base instead of the Madrid International Airport to avoid distorting the airport’s activities and hampering the evacuation operation.

The actions upon arrival were coordinated by the Ministry of Inclusion, Social Security and Migration (hereinafter referred to as the Ministry of Inclusion). Such actions included a systematic health control of the refugees and an assessment of their basic needs in terms of food and hygiene (Ministry of Health); identification procedures and security controls (Ministry of Interior); and an initial administrative processing of their host status (Ministry of Inclusion). All the actions were carried out in a 3000 m^2^ hangar with a capacity for up to 300 people.

When those actions were completed, the Spanish Red Cross transferred the refugees by bus to the so-called “Provisional Transit Facility” (PTF) located at the same base, with a capacity for up to 800 people in good conditions of habitability and sanitation. Two or three days later, the refugees were transferred to their final destination, either Spain or other countries.

### 2.2. Health Control System on Arrival

The implementation of the systematic health control system on arrival was the responsibility of Foreign Health. The DDFH developed a specific procedure under the coordination of the Directorate General for Public Health, which was an adapted version of the Foreign Health protocols available to respond to COVID-19 at Spanish points of entry. It was based on the legislation in force at that time, specifically the Resolution of 4 June of the Directorate General of Public Health on health controls at Spanish points of entry [5]. The new protocol was aimed at detecting people suspected of having COVID-19 or any other disease that could pose a public health risk and was designed as a dynamic procedure that could be modified according to the circumstances.

Health care, isolation and quarantine of refugees in case of need were also planned in coordination with the regional health authority of the Autonomous Community of Madrid. In this sense, a hospital was selected for health care and a medicalised hotel for isolation and quarantine.

Coordination between Foreign Health and the Autonomous Community of Madrid, as well as with the rest of the organisations involved, was one of the key elements of the response.

#### 2.2.1. Actions on Arrival

The actions implemented on arrival consisted of:

1. Temperature measurement after disembarkation and before entering the hangar;
2. Basic visual assessment for signs or symptoms of any infectious disease;
3. Rapid antigen test for COVID-19 in people over 12 years old, in accordance with Spanish regulations [5]. Testing of children under 12 years of age was only considered if they showed symptoms, if they were considered close contacts of a case or if at least 5% of positive cases were detected on their flight.

The mandatory Health Control Form that is usually required for all travellers entering Spain was not required for refugees for two reasons: first because the unstable situation they were living in their country made it impossible for them to obtain it in advance; and second because all of them travelled in the framework of an organised mission and had been previously identified.

#### 2.2.2. Human Resources

At least two Foreign Health teams composed of one medical doctor and one nurse from Madrid FHCU conducted the health control of each flight. The Spanish Red Cross (SRC) provided two more teams to help in flights where a larger number of passengers were expected.

In addition to this, supporting staff from the Ministry of Inclusion and the SRC were also present in the hangar to organise the spaces and manage the flow of people.

#### 2.2.3. Material Resources

A testing station for each health team was set up near the hangar door to maximise ventilation. Each station consisted of a main table to perform the tests and an auxiliary table with several chairs to accommodate the families that were going to be tested. The stations were equipped with infrared thermometers, hydro-alcoholic gel, biosafety containers and stationery.

The personal protective equipment used by the health control team consisted of FFP-2 masks (filtering face pieces), face shields, waterproof medical gowns and gloves. It was selected in accordance with the Procedure for Occupational Risk Prevention Services in the event of exposure to SARS-CoV-2 [6]. Panbio™ COVID-19 Ag Rapid Test Devices, included in the common list of rapid antigen detection tests for COVID-19 published by the European Commission on the basis of Council Recommendation 2021/C 24/01, were used to perform the tests [7].

#### 2.2.4. Procedure (Figure 2)

Families left the aircraft in groups. All people wore surgical masks, and new masks were provided to them on arrival in case of loss or damage during the flight.

**Figure 2:**
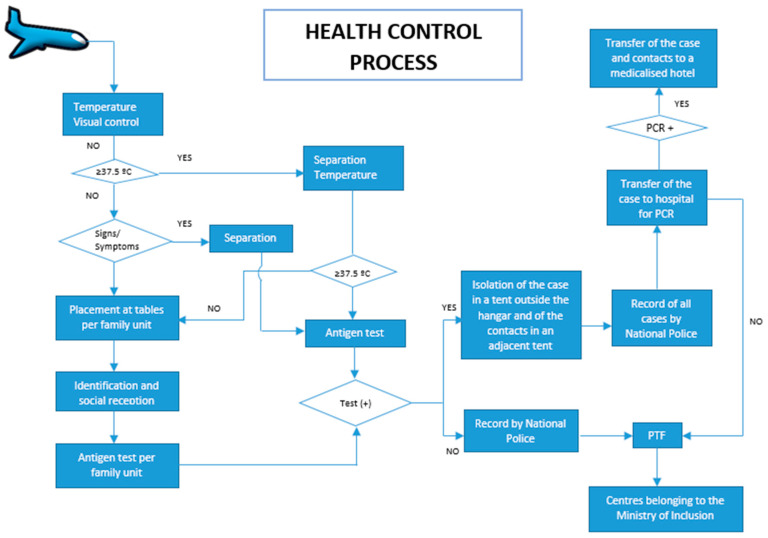
Procedure for health control on arrival. PCR: polymerase chain reaction. PTF: provisional transit facility.

At the hangar entrance, they were welcomed with the help of Afghan translators. A temperature measurement and a basic visual assessment for signs and symptoms suggestive of COVID-19 (cough, shortness of breath, odynophagia, headache or malaise) were carried out before entering the hangar. Individuals with a temperature of 37.5 °C or above or showing signs or symptoms were led to a specific area for further medical assessment and a new temperature measurement after five minutes. If the temperature remained high, the individual was tested immediately. If it had descended, he or she was reintegrated into their family unit.

All the families were accommodated at tables throughout the hangar so that each family occupied one of the tables.

When all the families were sitting at their respective tables, rapid antigen tests were performed. Test certificates were issued when needed.

Once the negative results of the tests were known, the families were accompanied to the National Police stations for registration and filiation. Finally, they were accommodated in the PTF to await their final destination, either the Spanish reception centres at the Autonomous Communities or other countries.

In the event of a positive test, the procedure provided that the person would be isolated in a tent outside the hangar, and their close contacts would be moved to an adjacent tent. For the purpose of this procedure, only the family members of a positive case were considered close contacts. The case would be transferred to the hospital for polymerase chain reaction (PCR) confirmation, and their close contacts would remain at the PTF. If COVID-19 was confirmed, all of them would be transferred to the hotel previously designated by the Autonomous Community of Madrid for isolation and quarantine, respectively. If COVID-19 was ruled out and the clinical situation of the case allowed it, he or she would be sent back to the PTF.

#### 2.2.5. Follow-Up in the PTF

Monitoring and health care of refugees at the PTF was carried out by the SRC in coordination with the DDFH and the Autonomous Community of Madrid. Antigen tests were performed on people presenting symptoms or if it was required by the countries of final destination.

Offering vaccination against COVID-19 to refugees in the PTF was considered but discarded. As refugees were leaving the PTF in 24–48 h, it was decided to offer them vaccination in their places of destination, following the Spanish COVID-19 vaccination strategy [8].

#### 2.2.6. Information Management Communications

The Ministry of Defense staff drew up a provisional list in Dubai with the names of the refugees travelling on each aircraft and forwarded it to the Ministry of Inclusion, which distributed it to the test stations.

Before starting the tests, each family was assigned to a specific test station to avoid confusion. Each station recorded the tests performed as well as the children under 12 years of age, noting a “T” or an “M”, respectively. Once all the families had been tested, the different lists were pooled so that a single list contained all the information.

At the end, the representative of the DDFH electronically sent a brief report to the Director General of Public Health and other parties. The report included the details of the flight (origin, day and time of arrival), the number of people controlled, the number of adults, the number of children under 12, the number of tests carried out, the results of the tests and any incidents that may have occurred.

## 3. Results

### 3.1. Flights

The operation took place between 19 and 27 August 2021. A total of 17 flights arrived with 2168 people, 680 of whom were under 12 years of age (Table 1). Eleven flights came directly from Dubai carrying refugees who would be staying in Spain. Another six flights had stopped over in other European countries (four in Paris, one in Rome and one in Athens) carrying refugees who would be transferred to other countries.

One of the flights from Dubai also carried 66 refugees who, as part of the NATO contingent, were bound for the United States. In this case, once the health control had been carried out, the US Embassy staff took responsibility for the rest of the administrative actions.

The arrival sequence of the flights varied. On 19, 22, 23 and 24 August, only one flight arrived; on 21 and 26 August, two flights; and on 20, 25 and 27 August, three flights. Flight occupancy was also variable, ranging from 35 to 94 people for those arriving from European airports and from 53 to 293 for those arriving directly from Dubai. The instability in Afghanistan and the difficulties inherent in the operation led to frequent changes, and flights could arrive at any time of the day or night.

### 3.2. Temperature Monitoring

Temperatures were taken in 100% of the refugees. Twenty-three of them were found to have a temperature ≥ 37.5 °C at the first measurement, which represents 1.06% of the total number of people monitored. In five of them (0.23%), a high temperature persisted after five minutes.

### 3.3. Initial Assessment of Signs and Symptoms

Eight persons (0.36% of the total) had mild symptoms during the initial assessment: cough, malaise and headache.

### 3.4. Rapid Antigen Tests

In total, 1499 rapid antigen tests were performed (Table 2), 1486 of which were performed in people over 12 years of age (100% of this group) and 13 in children under 12 years of age (1.62% of children under 12 years). One thousand four hundred and ninety-eight were negative and one positive in a 15-year-old girl presenting with a fever of 38 °C, malaise, headache, cough and dyspnea (0.067% of the total number of tests performed). The result was confirmed using PCR. Five close contacts were identified and quarantined at the designated hotel. None of them tested positive.

In addition, 68 more tests were performed at the PTF, either due to suspicion of the disease or if the country of destination required it. All of them were negative. That brought the total number of tests performed during the whole operation to 1567.

Seventeen people (0.78% of the total number of people monitored) were transferred to the hospital for diseases not linked to COVID-19:

- Two babies with gastroenteritis and dehydration;
- One baby with sunburn;
- Two boys with hyperglycaemia due to poorly controlled type I diabetes;
- Three boys with suspected chickenpox;
- One girl with suspected appendicitis;
- One male with shoulder trauma;
- One male with syncope;
- One female with absence seizures;
- One male with convulsive crisis;
- Two patients with arrhythmia;
- One female with psychiatric symptoms;
- It was also necessary to evacuate a 22-week pregnant woman for assessment.

## 4. Discussion

Under this operation, only one positive case was detected out of the 1499 tests carried out. This should not be surprising as it is consistent with the 14-day cumulative incidence reported by Afghanistan at the time of the evacuation (15.42 cases/100,000 inhabitants), despite a vaccination rate of just over 2% [9]. The early identification of the case allowed it to be handled immediately and probably prevented the spread of the disease, both among the people who were part of the operation and at the PTF, where the refugees lived very closely in a situation of high vulnerability.

As noted by the WHO and the European Centre for Disease Prevention and Control (ECDC), states have the obligation to protect and promote the right to health of all persons on their territory, including refugees and migrants [9,10]. The COVID-19 pandemic poses particular challenges for health protection in vulnerable settings, such as reception centres. It is important to assess the need to prevent and control disease transmission in those facilities, especially in the event of a sudden inflow of people [11,12] as in this case. The United Nations (UN) Inter-Agency Standing Committee (IASC) has developed helpful guidance for the prevention and control of COVID-19 in refugees and migrants in non-camp settings, such as points of entry or workplaces [13]. According to the ECDC’s recommendations at that time [14], reception centres for immigrants and refugees should be prioritised for testing due to the risk of the rapid spread of SARS-CoV-2 in such environments. All people with symptoms compatible with COVID-19 were to be tested, and cases not requiring hospitalisation were to be isolated or separated from other people in the centres’ facilities. Asymptomatic new arrivals were also to be tested to reduce the risk of introducing the disease into the centres, and the close contacts of identified cases were to be traced. All these measures were carried out during the reception process of refugees from Afghanistan.

In our case, the rapid development and immediate implementation of the health control procedure were possible because it was based on existing Foreign Health protocols that had been used at points of entry since the beginning of the pandemic [5]. One of the main elements of the protocol consists of organising the flow in an appropriate manner, as well as ensuring that all the staff have the same list of people to avoid the duplication of actions.

The measures adopted by the ministries involved in the response were agreed upon and approved by consensus within the Inter-Ministerial Commission, which was a guiding element that allowed operational planning. Under this framework, the planning, design and implementation of health control measures were coordinated between the DDFH and the Madrid FHCU under the supervision of the Directorate General for Public Health. Close collaboration with the SRC and the Autonomous Community of Madrid was also essential.

Since the Taliban’s seizure of power, the humanitarian situation in Afghanistan remains precarious and uncertain. According to the UN Secretary-General, the Asian country is facing a serious humanitarian, economic and political crisis [15]. In its report “The situation in Afghanistan and its implications for international peace and security”, the UN reports having received allegations of killings, enforced disappearances and other violations affecting the right to life and physical integrity of former government officials, security forces and people associated with the international military forces [15]. Similarly, the fundamental rights and freedoms of Afghan women and girls have been severely restricted. More than 24 million people are expected to be in need of humanitarian assistance in 2022, compared to 18.4 million people in 2021 [16,17]. In view of this situation, it is foreseeable that many Afghans will continue to try to flee their country in search of greater freedom and security. Measures such as the one we have taken in our country may therefore need to be repeated at different times and in different countries.

## 5. Conclusions

The global operation organised by Spain to evacuate people from Afghanistan was carried out successfully. The coordinated action of all the ministries involved in the response made it possible to manage the initial welcome of almost 2200 people, observing at all times guarantees of security, health and identification and offering them acceptable conditions of hygiene and health during their stay. Such a long-term intervention has never been undertaken in Spain, and there is no precedence at the Spanish border. Only the SRC has experience in providing humanitarian assistance to migrants entering Spain irregularly and assessing the risk of COVID-19 and other communicable diseases in reception centres for refugees, asylum seekers and migrants on behalf of the competent health authorities.

The health activities implemented as part of the global response were a real challenge for Foreign Health, taking into consideration the context of the pandemic. Foreign Health’s actions were decisive for assessing the health care of the refugees with the minimum risk to public health.

This event highlighted the importance of having an adequate health control system and a public health emergency response plan at points of entry to identify and manage people who may be infected. The system needs to be flexible enough to respond to unforeseen situations. Coordination between different health organisations involved in the response is a key element of the system.

Foreign Health already had a health control system at points of entry, which allowed measures to be implemented quickly. In addition, the excellent collaboration between health organisations made it possible to offer a comprehensive health response to the emergency.

Undoubtedly, the experience accumulated by Foreign Health in its more than 120 years of experience in health control at Spanish borders [18] has been essential in providing a rapid and high-quality response.

We consider that our experience in the response to this event could be useful for health professionals working in institutions in which they have to organise and coordinate a response to situations like this.

Finally, it should also be noted that Foreign Health personnel who participated in the “Antigone Operation” were awarded the Order of Civil Merit, an important national decoration whose purpose is “to reward the merits of a civilian nature of personnel dependent on any of the Public Administrations or by persons outside the Administration, who render or have rendered relevant services to the State, with extraordinary work, profitable initiatives, or with exemplary perseverance in the fulfillment of their duties” [19].

## Figures and Tables

**Table 1:** Flight information.

Flight	Arrival Date in Spain	Country of Origin	No. of Persons	No. ^1^ of Persons <12 Years Old
1	19 August 2021	Italy	37	13
2	20 August 2021	Dubai	53	5
3	20 August 2021	France	38	13
4	20 August 2021	Dubai	111	47
5	21 August 2021	France	35	7
6	21 August 2021	Dubai	113	31
7	22 August 2021	Dubai	177	52
8	23 August 2021	Dubai	260	78
9	24 August 2021	Dubai	293	115
10	25 August 2021	Dubai	140	51
11	25 August 2021	France	39	10
12	25 August 2021	Dubai	292	100
13	26 August 2021	Greece	94	25
14	26 August 2021	Dubai	241	56
15	27 August 2021	Dubai	139	45
16	27 August 2021	France	41	10
17	27 August 2021	Dubai	65	22
TOTAL			2168	680

^1^ No.: number.

**Table 2:** Information on tests performed, number of positives and close contacts identified.

Flight	No. Adults	No. Tests Performed Adults	No. Children <12 Years	No. Tests Performed Children <12 Years	Total No. of Tests Performed	No. of Positive Tests	No. of Close Contacts
1	24	24	13	1	25	0	0
2	48	48	5	0	48	0	0
3	25	25	13	0	25	0	0
4	64	64	47	0	64	0	0
5	28	28	7	1	29	0	0
6	82	82	31	0	82	0	0
7	125	125	52	6	131	1	5
8	182	180 ^2^	78	1	181	0	0
9	178	178	115	3	181	0	0
10	89	89	51	0	89	0	0
11	29	29	10	0	29	0	0
12	192	192	100	0	192	0	0
13	69	69	25	0	69	0	0
14	185	185	56	1	186	0	0
15	94	94	45	0	94	0	0
16	31	31	10	0	31	0	0
17	43	43	22	0	43	0	0
TOTAL	1488	1486	680	13	1499	1	5

^2^ Passengers exempt from test: one for absence crisis and one for epistaxis.

## Data Availability

Data sharing not applicable.

## References

[1] (1978). Spanish Constitution. “BOE” No. 311, 29 December 1978.

[2] (1986). Law 14/1986 of 25 April 1986, General Health. “BOE” No. 102, of 29 April 1986.

[3] (2011). General Law 33/2011 on Public Health. “BOE” No. 240, of 5 October 2011.

[4] World Health Organization (WHO) (2016). International Health Regulations (2005).

[5] (2021). Resolution of 4 June of the Directorate General for Public Health, Regarding Health Controls to Be Carried Out at Points of Entry into Spain; “BOE” No. 134.

[6] Spanish Ministry of Health Action Procedure for Occupational Risk Prevention Services in the Event of Exposure to SARS-CoV-2. https://www.sanidad.gob.es/profesionales/saludPublica/ccayes/alertasActual/nCov/documentos/Proteccion_Trabajadores_SARS-CoV-2.pdf.

[7] European Commission A Common List of COVID-19 Rapid Antigen Tests and a Common Standardised Set of Data to Be Included in COVID-19 Test Result Certificates. https://health.ec.europa.eu/health-security-and-infectious-diseases/crisis-management/covid-19-diagnostic-tests_en.

[8] Spanish Ministry of Health COVID-19 Vaccination Strategy: Updates. https://www.sanidad.gob.es/profesionales/saludPublica/prevPromocion/vacunaciones/covid19/Actualizaciones_EstrategiaVacunacionCOVID-19.htm.

[9] World Health Organization (WHO) (2020). Coronavirus Disease (COVID-19) Technical Guidance: Humanitarian Operations, Camps, and Other Fragile Settings as Well as Refugees and Migrants in Non-Humanitarian and Non-Camp Settings.

[10] Brandenberger J., Baauw A., Kruse A., Ritz N. (2020). The global COVID-19 response must include refugees and migrants. Swiss Med. Wkly..

[11] The European Asylum Support Office (EASO) (2020). COVID-19 Emergency Measures Asylum Reception Systems.

[12] Hoefer A., Despina Pampaka D., Daniel Castrillejo D., José Luengo-Cabrera J., Paisi M., Herrera-León S., López-Perea N., del Diego-Salas J. (2022). Considerations for COVID-19 management in reception centers for refugees, asylum seekers, and migrants, Spain 2020. Int. J. Infect. Dis..

[13] United Nation Office for Coordination and Humanitarian Affairs (OCHA), Inter-Agency Standing Committee (IASC) Inter-Agency Humanitarian Evaluation COVID-19 Global Humanitarian Response Plan: Learning Paper. https://interagencystandingcommittee.org/inter-agency-humanitarian-evaluations/inter-agency-humanitarian-evaluation-covid-19-global-humanitarian-response-plan-learning-paper.

[14] European Centre for Disease Prevention and Control (ECDC) (2020). Guidance on Infection Prevention and Control of Coronavirus Disease (COVID-19) in Migrant and Refugee Reception and Detention Centres in the EU/EEA and the United Kingdom—June 2020.

[15] United Nations (UN) (2022). The Situation in Afghanistan and Its Implications for International Peace and Security. General Assembly Security Council. https://undocs.org/es/S/2022/64.

[16] The UN Refugee Agency (UNHCR) Afghanistan Situation Update—15 January 2022. https://reporting.unhcr.org/document/1786.

[17] The UN Refugee Agency (UNHCR) (2022). Afghanistan Situation: Emergency Preparedness and Response in Iran. https://data2.unhcr.org/en/documents/details/90577.

[18] Madrid Gazette (1899). Reglamento de Sanidad Exterior (Foreign Health Regulations).

[19] (1998). Royal Decree 2396/1998, of 6 November 1998, Approving the Regulations of the Order of Civil Merit. “BOE” no. 279, of 21 November 1998.

